# Are you *how* you eat? Aspects of self-awareness in eating disorders

**DOI:** 10.1017/pen.2024.2

**Published:** 2024-05-21

**Authors:** Bruna de Moura Cortes Coutinho, Caio Gomes Pariz, Thomas E. Krahe, Daniel C. Mograbi

**Affiliations:** 1 Department of Psychology, Pontifical Catholic University of Rio de Janeiro. Rua Marquês de São Vicente 225 Gávea, Rio de Janeiro, RJ CEP 22451-900, Brazil; 2 Institute of Psychiatry, Psychology & Neuroscience, King’s College London, Psychology & Neuroscience, KCL, PO Box 078, De Crespigny Park, SE5 8AF, London, UK

**Keywords:** autobiographical memory, metacognition, self-awareness

## Abstract

Eating disorders (ED) are severe psychiatric disorders characterized by dysfunctional behaviors related to eating or weight control, with profound impacts on health, quality of life, and the financial burden of affected individuals and society at large. Given that these disorders involve disturbances in self-perception, it is crucial to comprehend the role of self-awareness in their prevalence and maintenance. This literature review presents different self-awareness processes, discussing their functioning across different levels of complexity. By deconstructing this concept, we can gain a better understanding of how each facet of self and personality relates to the symptoms of these disorders. Understanding the absence or impairment of self-awareness in ED holds significant implications for diagnosis, treatment, and overall management. By recognizing and comprehending the characteristics of self-awareness, clinicians can develop tailored interventions and evidence-based treatments for individuals with ED. Furthermore, this narrative review underscores the importance of considering temperament and personality factors in the context of ED, as temperament traits and personality characteristics may interact with self-awareness processes, influencing the development and maintenance of ED. Ultimately, the results highlight the pressing need for further research on the development of effective interventions and support strategies grounded in the aspects of self-awareness mechanisms for individuals affected by these disorders.

## 1. Introduction

Eating disorders (ED), psychiatric disorders characterized by dysfunctional behaviors related to eating or weight control (Treasure et al., 2020), can be extremely disabling, deadly, and costly, presenting a significant impact on the present and future health, as well as the quality of life of affected individuals, their caregivers, and society (van Hoeken & Hoek, 2020; Treasure et al., 2020; Smink et al., 2012). They also significantly impair physical health and disrupt psychosocial functioning (Treasure et al., 2020). In the USA, the total healthcare costs associated with ED were estimated to be $64.7 billion in the fiscal year 2018–2019, equivalent to $11 808 per affected person (Streatfeild et al., 2021). Therefore, it is evident that the impact of ED is substantial when considering mortality, disability, costs, quality of life, and family burden (van Hoeken & Hoek, 2020). This underscores the importance of gaining a better understanding of the psychological mechanisms and processes behind this condition.

According to the Diagnostic and Statistical Manual (DSM-5) and the International Classification of Diseases (ICD-11), there are six main ED: (i) Pica, (ii) Rumination Disorder, (iii) Avoidant/Restrictive Food Intake Disorder, (iv) Anorexia Nervosa, (v) Bulimia Nervosa, and (vi) Binge-Eating Disorder (Dawson-Squibb et al., 2023). With the exception of Pica and Rumination, which are considered more conceptual feeding disorders, these EDs are characterized by dysfunctional eating behaviors, excessive preoccupation with, and distress related to, body weight or shape, and specifically in the case of anorexia (AN), a persistent failure to recognize the seriousness of low body weight (Riva, 2014). Despite their unique characteristics, all ED significantly impact physical health and disrupt psychosocial functioning (Treasure et al., 2020). Epidemiological data indicate a heightened risk of mortality for all EDs (i.e., deaths per 1000 person-years), with 5.1 for anorexia and 1.7 for bulimia (Arcelus et al., 2011). One in five individuals with AN who died committed suicide (Smink et al., 2012). Moreover, resistance and denial are prominent features of these disorders, resulting in patients rarely seeking treatment voluntarily, which is largely true for AN (Riva, 2014). A distinctive characteristic of EDs is their ego-syntonic nature; patients suffering from such illnesses experience the disorder as part of their identity, thus highlighting the significance of self-awareness in EDs (Riva, 2014; Starzomska et al., 2018).

Self-awareness can be described as the ability through which individuals become the object of their own awareness (Morin, 2011). However, this definition suggests a unified model of the self, whereas in reality, it should encompass a range of processes (Mograbi et al., 2021). Consequently, self-awareness takes the form of a dynamic property consisting of diverse yet interconnected aspects. Mograbi et al. (2021, 2024) identify interoception, proprioception, agency, metacognition, emotion regulation, and autobiographical memory as components of self-awareness.

Considering that negative self-perception lies at the heart of ED, we believe that by delving into the various processes and aspects of self-awareness, we can gain a better understanding of how the disorder manifests and persists. Body image represents one of the primary concerns and focal points for these patients (Eshkevari et al., 2012). Perceptions, beliefs, and attitudes toward weight, body shape, and diet also play crucial roles, along with mechanisms of emotional dysregulation (Treasure et al., 2020).

The literature on ED already discusses these processes (e.g., Riva, 2014; Treasure et al., 2020; Martin et al., 2019; Palmieri et al., 2021). However, to the best of our knowledge, no study has yet summarized the relationship between ED and each of these components. Therefore, the present work aims to discuss the role of self-awareness in ED, considering the multiple processes associated with the dysfunctional features in these patients. Our objective is to delve deeper into self-awareness and gain a better understanding of how each component is affected in ED and how self-awareness is influenced at various levels and processes. This narrative review aims to provide valuable information about the extent and specificity of alterations in self-awareness within these clinical groups. Through this exploration, we aim to contribute to the advancement of effective approaches for individuals affected by these debilitating disorders. Additionally, recognizing and understanding changes in self-awareness can contribute to the clinical management of these conditions, as well as drive the development of new evidence-based treatments and interventions.

## 2. Self-awareness in Eating Disorders

Alongside the ego-syntonic nature of ED, we can also emphasize the allocentrism associated with this condition, that is the notion of the body as an object in the physical world. While an egocentric perspective frames the body as a first-person reference in one’s experience, ED patients often find themselves trapped in an allocentric representation of their bodies – a third-person perspective (Riva, 2012). In this view, individuals primarily rely on somatic representations, which encompass their understanding of the body as a continuous entity in the physical realm, whereas the egocentric frame is based on an integrated perception of their current bodily state (Riva, 2012; Galati et al., 2000; Longo et al., 2010). Furthermore, when locked into the allocentric frame, a negative body representation remains unaltered, as it is not updated by contrasting it with the perception from the egocentric frame. Although spatial experiences, including bodily awareness, typically involve the integration of various sensory inputs within both egocentric and allocentric frames, individuals with ED appear to have this system disrupted (Riva, 2012; Longo et al., 2010). The allocentric lock hypothesis seems to support the notion that self-awareness may be a core issue in ED.

As previously explained, self-awareness can be defined by multiple heterogeneous but interrelated processes (Mograbi et al., 2021, 2024). Since self-awareness encompasses several dissociable processes, understanding the particularities of each of them is crucial for comprehending the morbid changes observed in various psychiatric conditions. It is known that brain structural and functional alterations in individuals with clinical conditions can lead to variations in self-awareness processes (Bomilcar et al., 2021; Leslie et al., 2020; Marková et al., 2014). This notion has already been addressed in people with dementia, for example (Bomilcar et al., 2021; Mograbi et al., 2021), as well as in individuals with Bipolar Disorder (Silva et al., 2015) and other neuropsychiatric conditions (Mograbi et al., 2024).

In the case of ED, the clinical condition is characterized, and in some cases even defined, by changes in self-awareness. Therefore, it is essential to discuss their relationship with these multiple domains. This multifactorial perspective of self-awareness helps us understand differences between individuals with and without psychiatric diagnoses (Mograbi & Morris, 2014; Silva et al., 2015). Thus, it is understood that clinical conditions, such as ED, can result in selective deficits in self-awareness, compromising specific skills. In the following discussion, we will explore how impairments in self-awareness processes may underlie the development of characteristic symptoms of ED.

### 2.1 Interoception

A foundational aspect of self-awareness is interoception, which refers to the body’s ability to regulate its internal state through reflexes and adaptive responses. This process entails the regulation and stabilization of the internal physiological state of the body through homeostatic reflexes and adaptive, anticipatory (allostatic) responses. These responses are coordinated among organic systems and drive adaptive behaviors (Craig, 2002). From an evolutionary perspective, this foundational level is associated with the physiological conditions of the body, making it integral to the concept of self-representation (Damasio, 1999, 2010).

Being aware of stimuli and variations within the body allows us to perceive sensations such as thirst, nausea, the need to use the bathroom, hunger, and satiety. The transmission of information from the body to the brain occurs precisely to establish a dynamic and comprehensive representation of the physical self, which in turn coordinates behavioral responses to address these physiological needs (Craig, 2002). Although a significant portion of this process functions automatically through reflexes, the emergence of awareness initially stems from bodily sensations and emotional states. (Mograbi et al., 2021; Treasure et al., 2020). Hence, it becomes evident that the concept of body awareness encompasses interoception as a representation of the body’s internal state. This initial stage of awareness refers to the process by which the nervous system senses, interprets, and integrates signals originating from within the body, providing a moment-to-moment mapping of the body’s internal landscape across conscious and unconscious levels (Khalsa et al., 2018).

Three interoceptive skills have been suggested: accuracy, sensitivity, and awareness (Critchley & Garfinkel, 2017; Joshi et al., 2021). Interoceptive accuracy can be defined as the objective ability to detect internal states. Interoceptive sensitivity represents dispositional tendencies to be internally self-focused. Finally, interoceptive awareness indicates the level of self-knowledge, that is, the degree to which the subject perceives themselves capable of accurately judging their interoceptive sensations. Linked to these interoceptive abilities is an understanding of time. Time perception and estimation arise from the accumulation of bodily information and have been connected not only to interoceptive processes but also to subjective orientation, autobiographical memories, and emotion regulation (Richter & Ibáñez, 2021; Meneguzzo et al., 2022). Moreover, the impaired ability to estimate time in patients with ED seems to be related to compulsive self-monitoring (Meneguzzo et al., 2022). The overestimation of time in individuals with AN and the underestimation of time in those with BN can provide insights into the high levels of distress experienced by these patients (Holman & Grisham, 2020).

Following from that, hunger and satiety, which, as internal states of the body, also encompass accuracy, sensitivity, and awareness. Therefore, it is speculated how accurate, sensitive, and aware an individual with an eating disorder is. It is well known that dysfunctional processing of internal bodily cues related to hunger and satiety is a significant factor in disordered eating behaviors such as binge eating, purging, and cognitive restraint (Poovey et al., 2022). This interoceptive sensitivity presents itself as a crucial risk factor to address in screenings and interventions for disordered eating.

Recent research has identified a disturbance in the interoceptive system as a central mechanism underlying AN and BN. This disturbance affects various aspects of physiological condition interpretation, including hunger and satiety perception, risk prediction errors, emotional awareness, time accuracy, and body dysmorphia (Jacquemot & Park, 2020; Meneguzzo et al., 2022).

Furthermore, studies utilizing magnetic resonance imaging to measure postprandial gastric volumes have revealed that one in three AN patients experience feelings of fullness and lack of hunger even when their stomachs are empty (Bluemel et al., 2017). This suggests that disturbances in satiety perception in AN may arise from the interpretation and perception of visceral signals rather than differences in visceral sensitivity. Additionally, other modalities of interoception besides gastric distension may be implicated, as hunger ratings in AN patients have paradoxically decreased in response to insulin-induced hypoglycemia (Nakai & Koh, 2001). These misinterpretations of hunger and satiety signals could contribute to food avoidance and disrupted eating habits in AN (Jacquemot & Park, 2020).

Indeed, intuitive eating, characterized by the regulation of food intake based on visceral cues of hunger and satiety, correlates positively with interoceptive accuracy while showing a negative association with disordered eating patterns (DeVille et al., 2021). This ability to detect internal bodily cues, termed “interoceptive awareness,” along with the subsequent response to these cues, known as “interoceptive responsiveness,” plays pivotal roles in this process (Oswald et al., 2017). From this perspective, enhancing bodily cognition represents a crucial pathway toward reducing body misperception (Meneguzzo et al., 2023).

### 2.2 Proprioception and body ownership

Body awareness is guided by the representation of the physical extent of the body and the relative position of body parts in space, known as proprioception (Serino et al., 2013). Proprioception enhances the sense of owning a body and understanding its spatial location. Through this dynamic representation of proprioceptive information, one can become aware of their physical presence within a spatial context. Unlike the largely implicit nature of interoceptive information, proprioceptive information offers greater conscious access (Mograbi et al., 2021, 2024).

It is understood that body awareness results from the interaction between body image and schema, leading to a subjective sensation of one’s body (Thurm, 2012). The body schema allows individuals to recognize and perceive the various components of their own body, forming the perception of its dimensions regardless of body acceptance, self-esteem, or body type (Thurm, 2012). Perceived body image pertains specifically to an individual’s subjective perception or mental representation of their own physical appearance. In contrast, schema refers to broader cognitive frameworks that organize and interpret information across various domains, including beliefs, attitudes, perceptions, and expectations about oneself, others, and the environment (Young, 1999; Boone et al., 2013). It relies on multimodal sensory inputs, including exteroception, proprioception, vestibular, somatosensory, and visual systems (Riva, 2014). Regarding proprioception specifically, it is important to assess the degree of body distortion, perception of specific body segments, the sensory aspects of freely projected body perception, and the perception of body boundaries – with or without visual or cognitive interference, as well as any external reference (Thurm et al., 2011; Riva, 2014).

Based on clinical reports and a narrative review of the literature, it has been observed that patients with ED experience a distortion between their perceived body image and schema and their actual reality (Thurm, 2012; Miller, 1991; Longo & Haggard, 2012; Pellegrini et al., 2021; Riva, 2014). Consequently, individuals with ED exhibit a significantly stronger response to the rubber hand illusion, both in terms of perceptual (proprioceptive bias) and subjective (self-report) measures, compared to healthy controls (Eshkevari et al., 2014). This suggests that individuals with a history of ED may have heightened sensitivity to visual information about their bodies while exhibiting reduced processing of somatosensory information (Eshkevari et al., 2014).

Therefore, clinical interventions aim to reduce proprioceptive deviation, distortion, and dissatisfaction, while facilitating self-reintegration (Miller, 1991; Longo & Haggard, 2012). The interventions proposed to stimulate personal space align with the primary somatosensory representation involved in structuring body perception, which occurs through exteroceptive and proprioceptive stimuli (Longo & Haggard, 2012; Riva, 2014). These stimuli contribute to the formation of the body’s perceived boundaries. Since individuals with ED experience imprecision in perceiving their body dimensions, it is crucial to stimulate this representation to provide more accurate information about body dimensions to other cortical areas.

It is widely understood that our sense of self is closely related to both our physical bodies and our personal identity. Consequently, the formation and organization of our body image are integral aspects of our overall identity. From this perspective, the maintenance of an ED directly affects the subject’s identity (Pellegrini et al., 2021; Longo & Haggard, 2012). In line with self-objectification theory, which posits that individuals come to view themselves as objects to be evaluated based on appearance, the maintenance of an ED can exacerbate this tendency (Fredrickson & Roberts, 1997). Given that our understanding of our bodies plays a central role in assigning meaning to our experiences, addressing factors such as body interactions, sensorimotor patterns, and proprioceptive patterns is crucial in ED treatment (Pellegrini et al., 2021; Longo & Haggard, 2012).

### 2.3 Agency

Agency, or the sense of initiating our actions, plays a crucial role in self-awareness, often distinguishing clinical and non-clinical individuals (i.e., those not seeking medical or clinical treatment or evaluation). Action and agency are closely related, with the sense of agency encompassing self-efficacy through the awareness of deliberate control over one’s behavior. It involves recognizing intentional control over one’s actions (Haggard, 2017). When exploring this concept, the literature proposes a distinction between the feeling of agency and the judgment of agency. While the feeling of agency captures implicit, non-conceptual sensations of being an agent of action, explicit higher-level agency judgment is influenced by prior knowledge, expectations, and beliefs (Zapparoli et al., 2022). The latter aligns with metacognition and holds significance in psychotherapeutic treatment.

A sense of agency enhances our understanding of important processes in psychotherapy (Kristmannsdottir et al., 2019). In clinical psychology, agency has been defined as the ability to experience thoughts, emotions, and behaviors as belonging to the self and being under the influence and control of the self (Kögler, 2010; Williams & Levitt, 2007). The sense of agency can be measured in experimental settings by asking participants to explicitly judge whether their action caused an outcome event, or by using implicit measures, such as the compression of perceived time between action and outcome (Haggard, 2017). Thus, one can evaluate the concept of agency based on the degree to which a person believes they can impact their target experiences and behaviors (Colle et al., 2023). For instance, a patient’s sense of agency regarding symptoms of bulimia nervosa can be assessed concurrently with treatment (Kristmannsdottir et al., 2019).

Empirical findings can be assessed through different paradigms of implicit sense of agency in ED patients. Sensory attenuation, for example, consists of the fact that self-produced tactile stimulation is perceived as less intense than the same stimulus produced externally (Blakemore et al., 1999). This paradigm explains the difference between people’s intensity perception of tactile stimuli delivered by themselves (self-generated condition) and the stimuli delivered by other individuals (other-generated condition). This difference in perception gives rise to the so-called sensory attenuation phenomenon, in which the intensity of self-generated stimuli is perceived as significantly attenuated compared to the same stimuli generated by someone else. This phenomenon occurs because people automatically anticipate the sensory consequences of self-generated actions, explaining why people are unable to tickle themselves (Colle et al., 2023). Particularly, the first studies in this area show that in ED patients, there is an opposite pattern of behavior, with self-generated stimuli perceived as more intense than other-generated stimuli. This result indicates an alteration of the implicit component of the feeling of control in ED patients and alterations in the sense of agency (Colle et al., 2023).

Another paradigm is control feedback, which refers to a motivational factor: sensitivity to our degree of control over the environment (Eitam et al., 2013). When actions have even trivial and constant perceptual effects, motivation to perform is enhanced. Eitam et al. (2013) show that increased motivation is not explained solely by the availability of more information about task performance; rather, motivation increases only in conditions where control over the effects can be firmly established by the mind. Regarding EDs, it is known that the feeling of being out of control is present in AN and BN patients, especially in the latter (Adler et al. 2022; Brownstone et al., 2013). On the other hand, AN patients do not experience a self-perceived loss of control over eating (Adler et al. 2022; Brownstone et al., 2013). This may suggest that individuals with AN are aware that they can maintain control over eating, not least because they have restricted food intake resulting in significantly low body weight (Adler et al. 2022). Thus, AN behaviors may help maintain a sense of control and support the motivational sensitivity of the symptoms. Individuals with EDs appear to control their eating, weight, and shape as a way to address their perceived lack of control over interpersonal and overall life stressors, and these behaviors could be attempts at establishing control and managing internal uncertainty surrounding life events.

In relation to the need for control, we can also access agency by applying degrees of uncertainty (Perrykkad et al., 2021). It is known that uncertainty over actions negatively influences the sense of agency. Among all our sensations, we need to distinguish between those caused by ourselves and those caused by external factors (Perrykkad et al., 2021). Such paradigms, which modulate uncertainty, might provide more informative insights into the sense of agency in AN, as AN patients experience higher anxiety and a greater need for control in uncertain situations (Sternheim et al., 2011; Frank et al., 2012). The ability to infer agency is particularly challenging under conditions of uncertainty, and although the literature has not yet investigated this issue specifically in EDs, it could be a promising approach to exploring agency in this group.

Although the rubber hand illusion may offer greater insights into the proprioception domain, we can also evaluate the feeling of ownership of the fake hand. When AN patients experience a stronger sense of ownership over the rubber hand than healthy individuals (Keizer et al., 2014), we can begin to understand not only the intensity of the feeling of agency but also its accuracy. In this case, AN patients seem to have a false yet robust sense of agency (Colle et al., 2023).

The concept of agency can be particularly complex within the realm of ED. For instance, in AN, the need to maintain a sense of control is central (Kristmannsdottir et al., 2019; Engel et al., 2022). Patients with this diagnosis often perceive their symptoms as a solution to their problems (Kristmannsdottir et al., 2019; Bruch, 1973). Therefore, anorexic patients show a false yet powerful sense of agency (Colle et al., 2023). Conversely, individuals within the bulimic spectrum may experience a loss of control during compulsive episodes. In such cases, the sense of agency can be considered weak when the patient feels helpless or overwhelmed by ED-related emotions, thoughts, and behavioral symptoms (Kristmannsdottir et al., 2019). This dysregulation of the sense of agency occurs because ED symptoms often serve to shield individuals from feelings of inferiority and negative emotions (Kristmannsdottir et al., 2019). Gaining a deeper understanding of a patient’s agency can facilitate the adaptation of treatment approaches and methods that are best suited to their capacity for change. Consequently, enhancing the sense of agency may be an important objective in the treatment of ED (Engel et al., 2022; Colle et al., 2023).

### 2.4 Metacognition

Metacognition can be defined as the knowledge and cognitive processing involved in evaluating, monitoring, and regulating one’s own cognition (Flavell, 1979). It is a capacity observed, at different complexity levels, in a variety of species (Lage et al., 2022), being compromised in a number of neuropsychiatric disorders, such as obsessive-compulsive disorder and psychosis (Mograbi et al., 2024). Recent systematic review and meta-analysis articles have highlighted the association between ED and dysfunctional metacognition, specifically focusing on metacognitive beliefs and maladaptive metacognitive patterns such as rumination (Palmieri et al., 2021; Smith et al., 2018, Sun et al., 2017). In the context of ED, it is understood that dysfunctional metacognitions play a pivotal role in maintaining maladaptive coping strategies, thus contributing to the development and persistence of symptoms (Palmieri et al., 2021; Spada et al., 2008).

Rumination is considered a central feature in ED (Smith et al., 2018; Palmieri et al., 2021). In this metacognitive process, individuals repetitively focus on seeking out meanings, causes, and consequences of their negative emotions. This pattern of repetitive thoughts is configured as an amplifying cycle: rumination accentuates the negative mood state and, consequently, generates mood-congruent cognitions that increase repetitive thinking. This, in turn, reinforces and exacerbates the negative mood state (Nolen-Hoeksema et al., 2008). Importantly, patients with ED exhibit higher levels of rumination compared to healthy individuals. Furthermore, rumination is also associated with high levels of symptoms when only diagnosed patients are compared with each other (Smith et al., 2018). Difficulties in the capacity for cognitive flexibility have also been observed in this group of patients (Roberts et al., 2007; Tchanturia et al., 2004), emphasizing the relevance of repetitive thinking in the characterization of this condition. In the case of ED, rumination primarily manifests in relation to topics such as food, weight, shape, or physical appearance (Davenport et al., 2015). It has also been suggested that symptoms such as excessive focus on food and body weight and shape are actually forms of rumination specific to these disorders (Cowdrey & Park, 2011).

The occurrence and persistence of ruminative thoughts are associated with the metacognitive beliefs presented by an individual, as observed in both clinical and non-clinical samples (Palmieri et al., 2018). The main metacognitive beliefs observed in patients with ED include negative attributions to worries, such as the belief that worries are uncontrollable, lower levels of cognitive confidence (self-efficacy in decision-making and problem-solving), and higher levels of cognitive self-awareness, characterized by a tendency to focus on the self and monitor one’s thoughts (Smith et al., 2018; Palmieri et al., 2021). The belief regarding the need to control thoughts is also considered particularly relevant in the context of ED (Olstad et al., 2015). For instance, patients often express thoughts such as “I should always be in control of my thoughts” or “Not being able to control my thoughts indicates weakness.” These beliefs can be triggered by rumination frames, where the idea that “worries are unmanageable” arises when one finds themselves caught in a cycle of negative thoughts. Additionally, these beliefs can reinforce the repetitive thought pattern; for example, by believing that thoughts must be controlled, individuals reinforce the importance of constantly observing and analyzing them.

It is important to highlight the potential cyclical effect between maladaptive metacognitive beliefs and rumination. It is evident that rumination contributes to the consolidation of a negative mood state in this patient group, while also hindering engagement in more constructive forms of cognitive processing that could help regulate dysfunctional behaviors. Thus, taking a metacognitive perspective is essential when addressing ED.

### 2.5 Emotional regulation

There is an evident relationship between metacognitive processes and the ability to regulate emotions (Mograbi et al., 2021). The latter can be defined as the ability to modulate one’s affective experiences through cognitive, behavioral, intra-, and interpersonal strategies (Gross, 2002). Capabilities such as self-awareness of one’s emotions and the construction and execution of strategies aimed at goals and demands, necessary for adequate emotional regulation (Gratz & Roemer, 2004), involve metacognitive properties by definition. In fact, a direct association is observed between metacognitive beliefs, thought rumination, and emotional dysregulation, even in samples from the general population (Mansueto et al., 2022). This relationship is hypothesized to have reciprocal causality, meaning that beliefs, metacognitive processes, and psychopathological mood symptoms mutually affect each other (Capobianco et al., 2019).

Thus, it is not surprising that deficits in emotion regulation capacities are a central characteristic of ED (Ruscitti et al., 2016). A recent meta-analysis identified a strong association between maladaptive emotion regulation strategies and the symptomatology of such disorders (Leppanen et al., 2022). Interestingly, reduced use of acceptance, along with the adoption of strategies such as emotion suppression and avoidance, is also associated with dietary restriction habits in non-clinical populations (Mikhail & Kring, 2019). However, compared to psychiatric patients with other diagnoses, patients with ED face greater levels of difficulty with emotion regulation, including the ability to accept affective responses, achieve goals when affected by intense emotional states, and identify and understand their own emotions (Ruscitti et al., 2016).

In binge-eating disorder, emotional dysregulation, specifically difficulty controlling impulses, is associated with the severity of the episodes (Aloi et al., 2021). It is understood that this relationship is mediated by the ability to self-monitor, so difficulties with recognizing one’s own emotions play a key role in the development and maintenance of this disorder (Prefit et al., 2019; Westwood et al., 2017). An episode of binge eating can also be understood as a maladaptive emotion regulation strategy. In this sense, the ingestion of a large amount of food would be an attempt to alleviate negative feelings, but it would be dysfunctional as it causes new negative emotions at the end of the episodes (Leehr et al., 2015; Hilbert & Tuschen-Caffier, 2007). On the other hand, patients with anorexia nervosa or bulimia nervosa report feelings of pleasure or relief from implementing strategies such as food restriction or purging (Ruscitti et al., 2016). These behaviors can also be understood as attempts at emotional regulation. For example, food restriction to the point of causing starvation can reduce both the internal affective experience and the expression of emotions, helping the individual to avoid unwanted states (Leppanen et al., 2022). However, these strategies still constitute maladaptive approaches due to their resulting physical and emotional consequences. Despite possible distinctions in the specific skills and strategies within each condition, a meta-analysis found no differences in the existence and intensity of deficits in emotion regulation between the disorders. This finding reinforces the transdiagnostic nature of emotional dysregulation in eating disorders (Prefit et al., 2019).

In the realm of eating disorders (EDs), ambulatory assessment methods, particularly Ecological Momentary Assessment (EMA), have emerged as valuable tools for investigating the dynamic nature of ED symptoms in naturalistic settings (Maugeri & Barchitta, 2019). These methods allow researchers to examine the type, frequency, and temporal sequencing of symptoms as they occur in real-time (Smith & Juarascio, 2019). Additionally, there’s a growing interest in utilizing Ecological Momentary Interventions to target ED symptoms more effectively. These methodologies involve real-time data collection methods, based on repeated evaluations of emotions, behaviors, or experiences on a portable electronic device (e.g., a smartphone) throughout the day (at random or at specific moments) in natural environments (Walenda et al., 2021).

Negative emotions and deficits in their regulation are significant contributors to EDs, with increased negative emotions often preceding binge-eating episodes (Puttevils et al., 2021). Individuals with BED and BN experience heightened negative affect, particularly anxiety, and depression, leading up to binge-eating episodes, with fluctuations in mood and emotional states throughout the day often exacerbating those episodes (Walenda et al., 2021). EMA methodology research represents a way to focus on emotional changes related to the binge cycle, identifying its reinforcing factors, for instance.

Consistent with these observations, individuals with AN have demonstrated patterns of emotional dysregulation characterized by heightened negative affect, particularly anxiety and distress, preceding and following eating episodes or meal times (Puttevils et al., 2021). Individuals with AN often engage in maladaptive emotion regulation strategies such as avoidance or cognitive control, contributing to the maintenance of disordered eating behaviors (Wayda-Zalewska et al., 2022).

EMA studies can provide a wealth of data, including dietary, behavioral, physical, socio-psychological, and contextual information, facilitating the examination of concurrent exposures and events (Maugeri & Barchitta, 2019). These methods hold promise in various fields of nutritional epidemiology, from identifying determinants of dietary habits in healthy individuals to managing patients with eating or metabolic disorders. However, efforts to improve the validity, reliability, and technological innovations of EMA are encouraged for advancing public health research and interventions.

### 2.6 Autobiographical memory

Autobiographical memory is defined as the memory systems that encode, consolidate, and retrieve personal events and facts (Fossati, 2013). Because it is strongly related to self-representation, autobiographical remembering reflects an advanced state of consciousness that mediates awareness of the self as continuous across time (Levine, 2004). It also contributes to a sense of self, allowing autonoetic consciousness, time and travel, the ability to re-experience details, and therefore the development of self-knowledge (Lenzoni, Morris & Mograbi, 2020; Morris & Mograbi, 2013). In this context, a particularity of ED is the patient’s experience of identity (Riva, 2014).

Unlike many other conditions, EDs are also, to some extent, experienced positively by these patients (Vanderlinden, 2008; Serpell et al., 1999). This is because some of their maladaptive eating behaviors have social functionality, such as providing a sense of belonging, adequacy, control, and alignment with their belief system (Riva, 2014). Considering this direct relationship between the disorder and identity, it is relevant to discuss the association of the former with autobiographical memory, which plays a crucial role in the construction of identity and a sense of self (Wilson & Ross, 2003). In fact, different studies point to the existence of alterations in autobiographical memory processes in patients with ED. For example, patients with anorexia nervosa have been observed to have difficulties in accessing emotional memories (Ková et al., 2011), integrating positive and negative emotional experiences (Nandrino et al., 2006), and developing narrative discourse (Gandolphe et al., 2021). Additionally, these patients may exhibit a lack of specificity in autobiographical memories related to sensitive topics, such as food and the body (Terhoeven et al., 2023; Huber et al., 2015). They also report intense emotional activation and negative valence when recalling such experiences (Zhu et al., 2012). It is speculated that this overgeneralization of memories may serve as an avoidant regulatory mechanism (Oldershaw et al., 2015; Treasure & Schmidt, 2013). It is also known that overgeneral autobiographic memory disturbance is related to affective disorders and may represent a risk factor for depression, even due to possible comorbidity (Fang & Dong, 2022).

## 3. Personality factors

People with adaptive personalities appear to have positive views on many aspects of the self, and the other way around (Kamiya & Ito, 2000). There is a complex and multifaceted relationship between personality and ED (Barajas Iglesias et al., 2017; Dahl et al., 2012). Personality traits, temperament, and personality disorders are three main domains that have been investigated in this field (Rotella et al., 2016). Recent literature seems to confirm that specific personality and temperamental profiles can be drawn for these patients, which can help understand different diagnoses or symptoms (Barajas Iglesias et al., 2017; Petisco-Rodríguez et al., 2020; Rotella et al., 2016).

The Big Five theory suggests that there are five fundamental dimensions of personality that capture the most important aspects of individual differences (Smith et al., 2019). These dimensions are openness to experience, conscientiousness, extraversion, agreeableness, and neuroticism (DeYoung et al., 2016). Neuroticism refers to the tendency to experience negative emotions such as anxiety, sadness, fear, and irritability more frequently and intensely. People with expressive traits of neuroticism are often described as emotionally unstable, prone to mood swings, and easily stressed; this personality trait is defined as the propensity to experience negative emotions (Dahl et al., 2012; DeYoung et al., 2016).

People with high levels of neuroticism tend to be emotionally reactive and struggle with regulating their emotions (Yang et al., 2020). They frequently experience intense mood swings and often suffer from elevated levels of anxiety and worry (Hu et al., 2022). Moreover, they are more susceptible to feelings of fear and have an increased sense of threat perception (Carleton, 2016). As a result, individuals with neurotic tendencies are highly sensitive to stressors in their environment; they possess a lower threshold for perceiving situations as stressful and may find it challenging to cope with adversities (Hu et al., 2022). In addition to emotional regulation difficulties, these individuals commonly hold a negative self-perception. They tend to engage in self-criticism, exhibit low self-esteem, and frequently engage in excessive worry regarding their perceived shortcomings (Schmitz et al., 2003).

These characteristics may lead to susceptibility to psychological disorders, as they represent an increased risk of developing anxiety disorders (De Pasquale et al., 2022), depression (Lu et al., 2019), and somatic symptoms (Denovan et al., 2019; Macina et al., 2021). Neuroticism has been linked to a variety of factors, including genetics, early life experiences, and environmental influences (DeYoung et al., 2016; Frank et al., 2019; Hu et al., 2022). Understanding an individual’s level of neuroticism can provide insights into their emotional tendencies, coping mechanisms, and vulnerability to mental health issues (DeYoung et al., 2016).

In the context of ED, individuals with distorted perceptions of their own bodies may engage in self-deprecating thoughts, experience heightened levels of body dissatisfaction, and struggle with emotional instability (Hu et al., 2022; Mansueto et al., 2022; Schmitz et al., 2003). Positive associations between neuroticism and AN have already been demonstrated. However, despite the correlation between these two factors, causality cannot be inferred (Cassin & von Ranson, 2005; Bulik et al., 2002; Elfhag & Morey, 2008). Indeed, ego-syntonicity is more commonly associated with AN, whereas it is less prevalent in BN and BED.

Psychological distress and maladaptive coping skills may also mediate the relationship between neuroticism and ED (He et al., 2019). Unlike neuroticism, which is a personality trait, psychological distress is an emotional state and is much more amenable to change through appropriate intervention (He et al., 2019; McAdams, 1992).

Thus overall, the study of personality in eating disorders has revealed significant clinical implications, as treatment predominantly relies on psychotherapeutic interventions. Furthermore, the inclusion of personality factors in the etiology and maintenance of eating disorders underscores the importance of accentuating the examination of metacognition in these particular patients.

## 4. Neural correlates

Neural correlates of interoception appear to be associated with regions such as the insula, subcortical regions, and brainstem regions, all associated with homeostatic processes (Mograbi et al., 2021; Kim et al., 2012). Supporting these findings, a review has highlighted the connection between areas of the anterior cingulate and insula and an individual’s orientation toward eating behaviors (Frank et al., 2019). While the anterior cingulate areas are involved in regulating emotions, cognitive processes, decision-making, and error detection, the insula is known for processing and integrating sensory information, emotional responses, and interoceptive processes (Aouizerate et al., 2007; Uddin et al., 2017). According to this review, patients diagnosed with anorexia demonstrate greater connectivity between the dorsal anterior area (which typically functions involve cognitive control, attention regulation, and response selection) and the posterior cingulate gyrus (involved in self-reflection, memory retrieval, and processing emotional stimuli) (Rolls, 2019; Lee et al., 2014). In contrast, patients diagnosed with bulimia show enhanced connectivity between the dorsal anterior cingulate cortex (conflict monitoring) and the medial orbitofrontal cortex (reward processing and emotional regulation) (Frank et al., 2019; Heilbronner & Hayden, 2016; Loh & Rosenkranz, 2023). One study compared women diagnosed with anorexia, weight-restored women with anorexia, and controls (Holsen et al., 2012). In comparison to controls, both weight-restored women and those with active disease demonstrated pre-meal hypoactivity in the anterior insula in response to high-calorie foods. Reduced neural activity in the anterior insula may indicate diminished responsiveness to stimuli, potentially impacting emotional and interoceptive processing (Yoon et al., 2015; Gray & Critchley, 2007). After the meal, hypoactivation in the anterior insula persisted in women with active disease (Holsen et al., 2012). These patterns suggest dysfunctionality of the circuit in question, possibly predisposing the observed behaviors in eating disorders.

Thus, the behavioral changes observed in these conditions are correlated with functional changes in brain circuits and connectivity associated with reward, habit learning, interoception, and the construction of the self in general (Frank et al., 2019; Treasure et al., 2020). These findings support the idea that there is indeed a relative imbalance between appetite regulation, in line with impairment in interoception (Frank et al., 2019). The perception of being fat while being severely underweight may have its foundation in abnormal neurocircuitry of interoception, in addition to cognitive-emotional processes.

In terms of emotional processes, it is understood that body dissatisfaction plays a significant role as both a precipitating and maintaining factor. Behavioral studies indicate that a cognitive-affective and perceptual component (involving perceptual disturbance of the body itself) is crucial in understanding this pathophysiology (Friederich et al., 2010). Although the functional neuroanatomy underlying body dissatisfaction in AN remains inadequately understood, some empirical studies have begun to explore its neural correlates. Functional magnetic resonance imaging (fMRI) studies suggest the activation of the right sensorimotor regions of the brain alongside decreased activation of the rostral anterior cingulate cortex (ACC) (Friederich et al., 2010). The hyperactivation of the insula coupled with hypoactivation of the ACC may play a critical role in distorting interoceptive awareness related to self-comparisons of the body and/or altering implicit motivation for idealized body comparisons (Mograbi et al., 2021; Friederich et al., 2010). Conversely, impaired inhibitory control is recognized as a behavioral phenotype in patients with BN. Consistent with this notion, diminished activation is observed in the right sensorimotor area (postcentral gyrus, precentral gyrus) and the right dorsal striatum (caudate nucleus, putamen) (Skunde et al., 2016). The reduced activation within the frontostriatal brain circuitry in individuals with bulimia nervosa is believed to contribute to the severity of binge-eating symptoms (Thomas et al., 2022).

Furthermore, individuals with AN commonly encounter challenges in social interactions, which could be linked to the heightened activity of brain regions including the right temporoparietal junction, the bilateral medial prefrontal cortex, the cerebellum, and the dorsolateral prefrontal cortex (Leslie et al., 2020). These regions are notably associated with aspects of self-awareness such as metacognition, emotion regulation, and autobiographical memory (Mograbi et al., 2021). The difficulties in socio-emotional processing seen in AN might stem from reduced connectivity between the bilateral occipital facial area and various brain regions including the cingulate, precentral, superior, middle, medial, and inferior frontal gyri, especially when compared to individuals without the disorder (Halls et al., 2021).

In a recent study, patients with AN, compared to healthy controls, exhibited less activation in areas such as the precuneus and angular gyrus, associated with self-referential processes, when performing tasks involving recalling autobiographical memories related to food and the body. However, no differences were observed when evoked memories were neutral (Terhoeven et al., 2023). These findings not only indicate changes in the autobiographical process of these patients but also support the role of suppressing and avoiding emotions in maintaining the condition. This concept is also related to the development of self-knowledge and therefore implicated in personality identity construction (Wilson & Ross, 2003).

It is important to recognize that brain functions are part of a complex network of interconnected brain regions, and the boundaries between specific areas are not rigid. There are overlaps and interactions between different regions, highlighting the intricate nature of neural processing in the brain.

Despite the limited understanding of the neural mechanisms underlying ED, their intersection with brain areas linked to self-awareness is evident. Approaching the study of ED through the lens of self-awareness offers promising avenues for the development of novel treatment models and interventions in psychotherapy. Understanding the neural correlates of EDs is crucial for unraveling the complex interplay between brain function and the manifestation of these psychiatric conditions. Research in this area aims to identify brain regions, circuits, and neurobiological mechanisms contributing to the development, maintenance, and progression of the disorders. By examining neural correlates, researchers seek to elucidate how alterations in brain structure and function may underlie the cognitive, emotional, and behavioral dysregulation observed in individuals with EDs.

## 5. Discussion

Based on the revised concepts, the present work aimed to discuss the relationship between ED and the multiple components of self-awareness. In general, this condition is characterized by the interaction between cognitive, socio-emotional, and interpersonal elements (Riva, 2014). While various studies examine self-awareness in these disorders focusing on specific dimensions such as metacognition, emotion regulation, or self-image, it is evident from the present review that psychopathological models of ED should incorporate the multiple components and dynamic processes of self-awareness. Therefore, developing explanatory models and testing hypotheses related to these symptoms’ manifestations can be a promising future direction in investigating the relationship between self-awareness and ED.

Moreover, understanding this relationship can greatly impact the development of therapeutic treatments. Despite advancements in clinical research on ED, an increasing body of literature indicates that patients often do not receive evidence-based treatment (Hilbert & Tuschen-Caffier, 2007). In terms of psychotherapies, Cognitive Behavioral Therapy (CBT) has demonstrated more significant improvements in symptomatology among individuals with ED (Hay et al., 2014; Lammers et al., 2020; Treasure et al., 2020). Within the scope of intervention options for EDs, CBT was compared with various other therapeutic modalities, including Behavioral Systems Family Therapy, Interpersonal Psychotherapy, Psychodynamic Psychotherapy, Group Therapy, Occupational Therapy, Psychoanalysis, Cognitive Analytic Therapy, Focal Psychoanalytic Therapy, and other psychodynamic therapies. For more comprehensive insights, please refer to the review conducted by Hay et al. (2014). However, according to the National Institute for Health and Care Excellence (NICE, 2020), even CBT strategies can sometimes prove ineffective and, even when successful, carry a high risk of relapse. Despite these challenges, it remains unclear which factors mitigate the risk of relapse following successful treatment or the benefits individuals receive from additional treatment to prevent relapse (NICE, 2020; Vanderlinden, 2008). Moreover, there is limited evidence regarding effective relapse prevention strategies for individuals in remission (NICE, 2020). In this regard, comprehending the mechanisms of self-awareness associated with these disorders assumes greater importance for the clinical treatment of these conditions. In interim treatment, especially for children and adolescents struggling with severe manifestations of the disease, a multidisciplinary approach should be adopted. This involves integrating targeted psychological therapy, such as CBT, to address the eating disorder and concurrent psychological issues. A comprehensive treatment plan should include psychoeducation for families, nutritional and medical interventions, occasionally incorporating pharmacotherapy. In some cases, it may require collaborative case management with schools and other relevant agencies (Hay et al., 2014).

This narrative review aimed to delve into the study of self-awareness within eating disorders, exploring its multifaceted aspects. It is crucial to note, however, that this review was not intended to be exhaustive, and we acknowledge the possibility that some relevant studies may have been omitted. Therefore, it is important to emphasize that the findings presented should be interpreted within the confines of this scope, recognizing the existence of potential gaps that may influence the conclusions drawn.

Embracing this comprehensive perspective of self-awareness is pivotal for gaining a deeper understanding of eating disorders and for the development of future effective interventions. By employing a dimensional approach to EDs, wherein management strategies are customized according to the stage of illness severity and the individual’s symptom profile, we hope to optimize treatment outcomes. This underscores the importance of conducting detailed evaluations of self-awareness and providing psychoeducation to healthcare professionals, patients, and their families. We aspire that addressing these facets of self-awareness will pave the way for the development of new therapist-led manualized approaches and interventions.
